# A Case of Elephantiasis—Successful Recovery

**Published:** 1867-01

**Authors:** R. Dexter

**Affiliations:** Chicago


ARTICLE VII.

### A CASE OF ELEPHANTIASIS—SUCCESSFUL RECOVERY.

BY R. DEXTER, M.D., Chicago.

Carrie G., *æt.* 20, an inmate of the Erring Women's Refuge, gives the following history of herself and the outgrowth:—

Is a native of Rochester, N.Y.; has resided in the southern part of Illinois several years. A little more than a year ago, she observed that the external labia were somewhat increased in size, and presented an unusually rough appearance. The rapid growth of the parts induced her to seek medical advice. The external application of iodine was prescribed, together with the exhibition of internal remedies, but without any beneficial results.

The advice of another physician was sought, but change of treatment brought no relief.

Our attention was called to the case about two months since. Found the external labia enormously hypertrophied, presenting every appearance and symptom of elephantiasis. Each lip was about five inches long, and one and a-half inches through. It seemed that but one alternative remained, *viz.:*—excision. The counsel of a medical gentleman was obtained, who fully concurred in the opinions above given.

Accordingly, on the 21st of December last, we removed the entire mass. Hemorrhage was controlled during the operation by tying the arteries as they were divided. There were four on each side, but were not enlarged. Recovery will be perfect, with but trifling deformity.

